# Combination therapy of 7-O-succinyl macrolactin A tromethamine salt and temozolomide against experimental glioblastoma

**DOI:** 10.18632/oncotarget.23295

**Published:** 2017-12-14

**Authors:** Jun Jin, Kihwan Hwang, Jin-Deok Joo, Jung Ho Han, Chae-Yong Kim

**Affiliations:** ^1^ Department of Neurosurgery, Seoul National University Bundang Hospital, Seongnam-Si, Korea; ^2^ Department of Neurosurgery, Seoul National University College of Medicine, Seoul, Korea

**Keywords:** glioblastoma, migration, invasion, macrolactin A, SMA salt

## Abstract

7-O-succinyl macrolactin A has shown anti-inflammatory, anti-angiogenesis, and anti-metastatic effects. It also exhibits strong suppression of tumor growth. In our previous study, we assessed the anti-neoplastic effects of 7-O-succinyl macrolactin A tromethamine salt (SMA salt) on a glioma cell line. Moreover, according to our data, SMA salt might be contributed to the inhibitory effects on migration and invasion, as well as a cytotoxic effect on the glioblastoma cell lines. In the present study, we investigated the anti-tumor effects of combination therapy with SMA salt and temozolomide (TMZ) in glioblastoma cell lines. The combination therapy affected cell viability significantly, decreasing in glioblastoma cell lines. In cell migration assays, combination therapy showed more inhibitory effects than TMZ in these cell lines. The tumor volume was significantly decreased in the combination group compared with both TMZ and control groups by using the orthotopic mouse model. The effects of combination therapy with SMA salt and TMZ attributed to the inhibition of migration, invasion activities and anti-tumor effects. SMA salt could be one of the promising candidates for combination therapy in clinical settings.

## INTRODUCTION

Glioblastoma (GBM) is the most frequent primary malignant brain tumor in adults [1, 2]. Although GBM patients receive aggressive multi-disciplinary treatments such as surgery, radiation, and chemotherapy, their median survival is less than 15 months [3–8]. In 2005, Stupp et al. reported that median survival was longer in the radiation plus temozolomide (TMZ) group than the radiation only group. Many researchers were concerned about the effect of TMZ on GBM patients or combination effects with other drugs. Therefore, numerous articles were published associated with TMZ.

TMZ is a well-known alkylating agent and is applied widely with GBM as a first-line treatment [9–11]. TMZ is rapidly absorbed with almost 100% bioavailability by oral administration [12, 13]. It crosses the blood-brain barrier and achieves effective concentrations in the CNS, approximately 30% to 40%. However, treated with highly anticipated TMZ, most GBM patients still die within 2 years. It is important to find a new regimen to prolong the survival of GBM patients.

In recent years, the anti-angiogenic agent has gained popularity from researchers and physicians. Some groups worked on anti-angiogenic agents, such as cilengitide or bevacizumab [14–17]. However, these papers did not produce desirable results. In a randomized phase 3 study with bevacizumab, the progression-free survival was longer in the bevacizumab group (10.6 months) than in the placebo group (6.2 months), whereas the overall survival was similar in both the bevacizumab and placebo groups (16.7 months) [14]. In another multicenter, randomized, phase 3 study, there was no significant difference in overall survival between the control group (26.3 months) and the cilengitide group (26.3 months) [15]. This study only included O6-alkylguanine DNA alkyltransferase (MGMT) promoter methylated patients, thus the overall survival was longer than other studies.

7-O-succinyl macrolactin A (SMA) and macrolactin A (MA) are macrolactins generated from *Bacillus polyfermenticus* KJS-2 [18]. Macrolactins are macrolides containing three separate diene structure elements in a 24-membered lactone ring [19]. Macrolactins were first described in 1989 by Gustafson et al. and were reported to have antiviral properties, with MA being the most active compound of the group [19]. Then, SMA was found in 2000 by Jaruchoktaweechai et al. [20], and Romero-Tabarez et al. also described the inhibition of vancomycin-resistant enterococci in 2006 [21]. According to reports, SMA has shown anti-angiogenic and anti-metastatic effects [22, 23]. In our previous study, we assessed the antineoplastic effects of 7-O-succinyl macrolactin A tromethamine salt (SMA salt) on GBM. Moreover, SMA salt might attribute to the inhibitory effects on migration and invasion, as well as cytotoxic effects on glioma cell lines [24].

In the present study, we investigated the anti-tumor effects of combination therapy with TMZ and SMA salt in GBM cell lines.

## RESULTS

### GBM cell line sensitivities to SMA salt and TMZ

**Figure 1:**
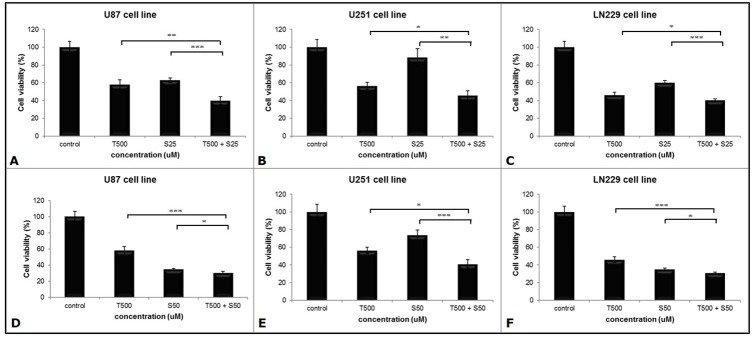
The anti-tumor effect on the viability of the different cell lines Three cell lines were seeded in 96-well-plates and treated with SMA salt only, TMZ only, or a combination therapy. Cells were treated with both 25 μM **(A, B** and **C)** and 50 μM **(D, E** and **F)** concentrations of SMA salt. ^*^P < 0.05, ^**^P < 0.001, and ^***^P < 0.0001.

We assessed the effects of combination therapy on GBM cell lines for 48 hours. In the U87 cell line, the viability of the combination group (39.9%) was significantly lower than other two groups (TMZ group, 58%; SMA salt group, 62.8%) at the concentration of SMA salt at 25 μM (P < 0.001 and P <0.0001). In U251 and LN229 cell lines, the combination groups (46% and 40.3%) also showed significantly lower cell viability than the TMZ group (56.5%, P < 0.05 and 46.1%, P < 0.05) and SMA salt group (88.4%, P < 0.001 and 59.9%, P < 0.0001) at a 25 μM concentration of SMA salt (Figure 1A–1C). Moreover, at the 50 μM concentration of SMA salt, the cell viability of each cell line was similar to the 25 μM concentration of SMA salt (Figure 1D–1F). We found that there was no significant change when the concentration of SMA salt was doubled in the U251 cell line. However, in the other two cell lines, a doubled concentration of SMA salt resulted in approximately half the decrease in cell viability.

### The effects of SMA salt and TMZ on migration and invasion

**Figure 2:**
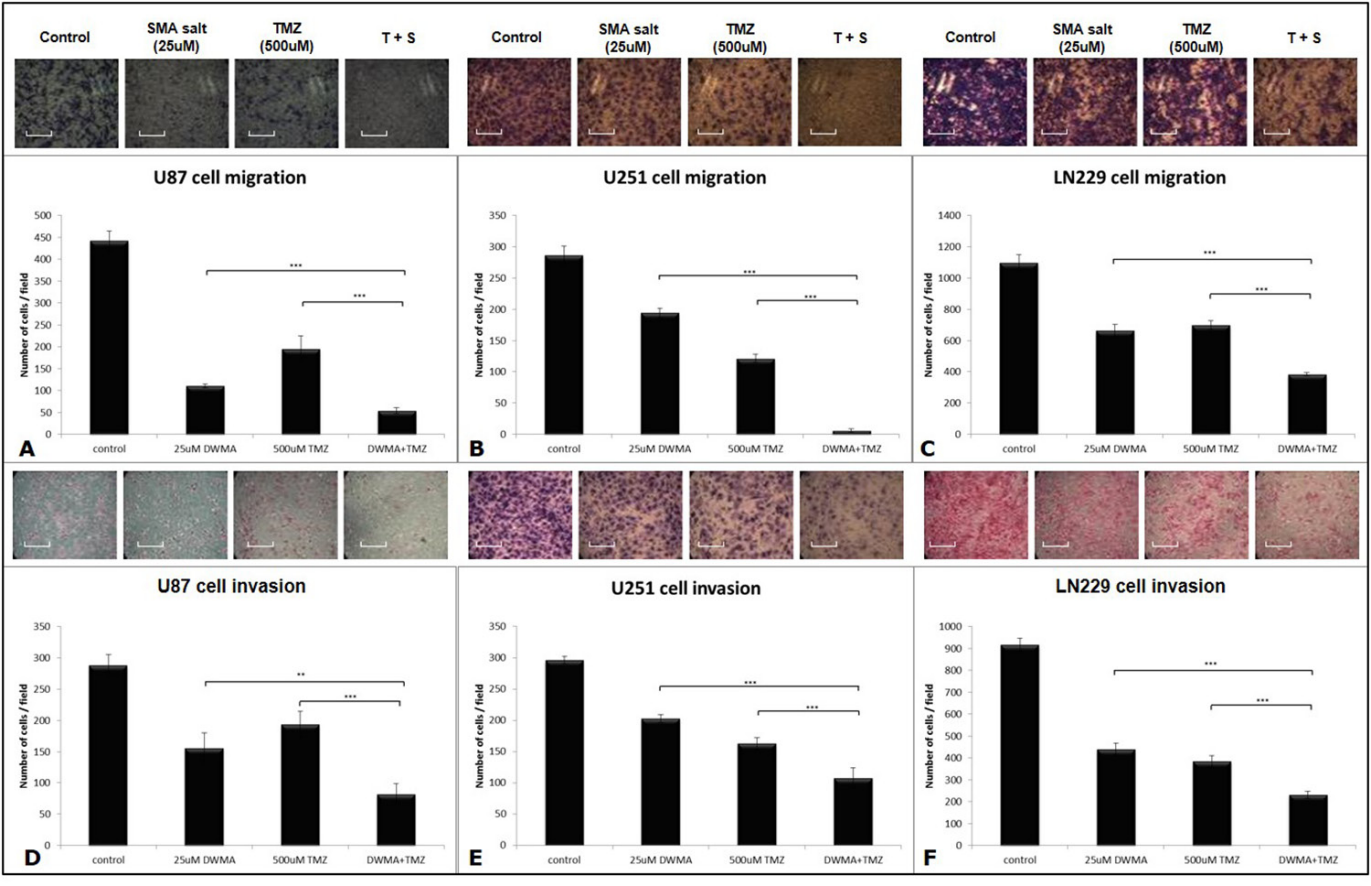
Cell migration and invasion assay of U87, U251, and LN229 cell lines The 25 μM concentration of SMA salt was chosen to perform the cell migration **(A, B** and **C)** and invasion **(D, E** and **F)** assays with the Transwell apparatus. The scale bar on the image is 35.29 μm. ^*^P < 0.05, ^**^P < 0.01, and ^***^P < 0.001.

We performed the cell migration assay with the 24-well Transwell apparatus and followed the manufacturer's protocol. Eventually, the stained migrating cells were counted to assess the effects of SMA salt and TMZ. The cell migration abilities in U87 cell lines were significantly decreased in the combination group (12.2%) than in the SMA salt (25.03% and P < 0.001) and TMZ groups (44.01% and P < 0.001) (Figure 2A). Similar results were found in the LN229 cell line. There was a significant difference among the combination group (35.1%), SMA salt group (60.49% and P < 0.001) and TMZ group (63.72% and P < 0.001) (Figure 2C). Moreover, the inhibitory effect of migration on the U251 cell line was significantly higher in the combination group (8.17%) than in the TMZ group (42.38% and P < 0.001) and SMA salt group (67.9% and P < 0.001) (Figure 2B). However, The U251 cell line showed a different pattern with U87 and LN229 cell lines.

We also evaluated the inhibitory effect of invasion in three cell lines, U87, U251, and LN229. The combination group showed greater inhibitory effects than the SMA salt and TMZ groups on cell invasion in the three cell lines (Figure 2D–2F). In the U87 cell line, the combination group (28.45%) showed greater inhibitory effects when compared with the SMA salt group (53.99% and P < 0.01) or TMZ group (67.24% and P < 0.001). In the U251 cell line, there was a significant difference in the combination group (36.55%) compared with the SMA salt (68.45% and P < 0.01) or TMZ groups (55% and P < 0.05). Similar results were also shown in the LN229 cell line. The combination group (25.26%) showed a significant difference not only in the SMA salt group (48.13% and P < 0.001) but also in the TMZ group (42.01% and P < 0.01). Interestingly, the U87 cell line showed results in both the cell migration assay and invasion assay. Moreover, the same situation was observed in the other two cell lines.

### The effects of SMA salt and TMZ on tumor volume and survival analysis

**Figure 3:**
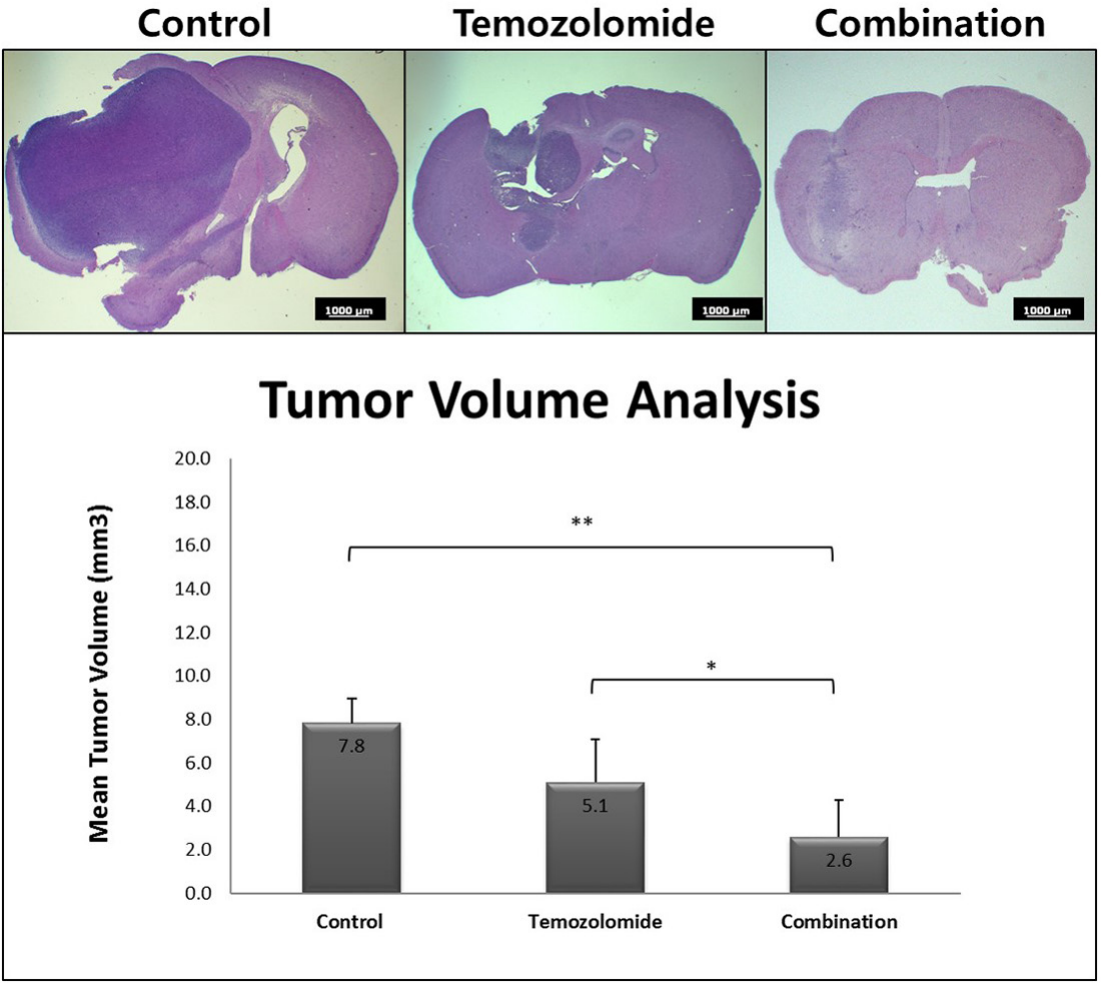
The change of tumor volume was due to treatment with temozolomide only and the combination therapy The mean tumor volume of the combination treatment group was significantly lower than the control group (^**^P < 0.01) and temozolomide only treatment group (^*^P < 0.05). The scale bar on the image is 1000 μm.

The inhibitory effects on tumor growth were evaluated by tumor volume assessment. To mimic the clinical situation, mice grew tumors for 1 week. After 1 week of the stationary phase, they were administered treatment for 4 weeks with proper drugs, including TMZ or SMA salt. We obtained the mean tumor volume from each group. The mean tumor volume in the combination group (2.6 ± 1.7 mm^3^) was significantly smaller than in the TMZ group (5.1 ± 1.9 mm^3^, P < 0.05) and the control group (7.8 ± 1.1 mm^3^, P < 0.01) (Figure 3).

**Figure 4:**
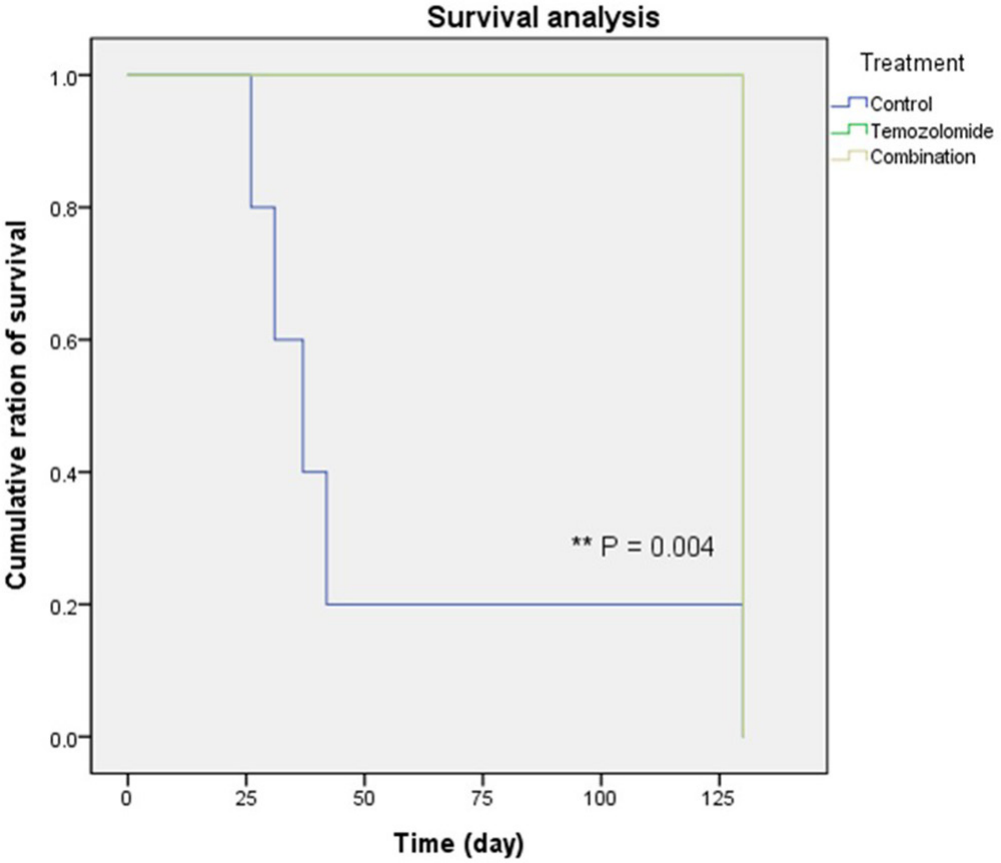
Survival of the glioma xenograft mouse model Most of the control group mice (blue line) died within 40 days. However, mice in the remaining groups (green and yellow lines, two lines were overlapped) lived to the end of the experiment. In addition, there was no difference between the temozolomide only and combination treatment groups.

The data in Figure 4 includes the survival analysis on the orthotopic mouse model. The mice were divided into three groups: a control group, TMZ group, and combination (TMZ and SMA salt) group. The mean survival period of the control, TMZ, and combination groups was 53 days, 130 days, and 130 days, respectively. The overall survival in the TMZ group was significantly higher than in the control group (P = 0.004). In addition, overall survival in the combination group was also significantly higher than in the control group (P = 0.004). However, the data showed that there was no significant difference between the TMZ group and combination group.

## DISCUSSION

SMA and MA are macrolactins generated by *Bacillus polyfermenticus* KJS-2 [18]. Both are well known as antibacterial substances [18, 25]. According to other research, SMA has better antibacterial activity and less cytotoxicity than MA [26]. SMA has also shown other effects such as anti-inflammatory, anti-angiogenesis and anti-metastatic effects [22, 23, 26]. Moreover, Sushil C. Regmi et al. used colon and lung cancer xenograft models to prove the anti-tumor effect and suggested that SMA could function as a potential monotherapy or in combination with the cytotoxic chemotherapeutic drugs [27]. In our previous study, we assessed the anti-neoplastic effects of SMA salt on GBM cell lines [24]. Moreover, according to our data, SMA salt might attribute to the inhibitory effects on migration and invasion and had cytotoxic effects on the GBM cell lines. Ultimately, we suggest SMA salt as a monotherapy with anti-tumor activity in a glioma orthotopic mouse model.

In the present study, we focused on investigating the anti-tumor effects of combination therapy with SMA salt and TMZ in GBM cell lines. In this study, we used previous experience to assess the combination therapy with SMA salt and TMZ. The data showed that the GBM cell viability, which was used in this study, was lower in the combination therapy than in the TMZ group. We also found the same situation in the migration assay and invasion assay. There was an interesting finding that U87 and LN229 cell lines were more sensitive than the U251 cell line when treated with a doubled concentration of SMA salt. From the *in vitro* data, we suggest that combination therapy with SMA salt and TMZ may attribute to the anti-tumor effect on the GBM cell lines.

The orthotopic mouse model was used to evaluate the effect of combination therapy with SMA salt and TMZ. The implanted tumor volume that was obtained from each orthotopic mouse was calculated to compare the mean tumor volume of three groups. The mean tumor volume in the combination group was lower than in the TMZ group and control group.

Four-month-long survival analysis was carried out simultaneously with mean tumor volume evaluation. In this experiment, the overall survival in the combination and TMZ groups was longer than in the control group, respectively (P = 0.004, and P = 0.004). However, there was no significant difference between the combination group and TMZ group. We assumed that the main reason there was no difference between the two groups was due to the blood-brain barrier (BBB) and time. SMA salt may or may not cross the BBB. To discover this, we would like to do more research on the BBB and SMA salt. Furthermore, we should prolong the period of survival analysis to observe the difference between the temozolomide group and the combination group. Overall, the *in vivo* experiment data indirectly demonstrated the effect of combination therapy in the orthotopic mouse model with the U87 cell line.

Another strong effect of SMA was anti-angiogenesis. Anti-angiogenesis was explored in publications by Judah Folkman in the 1970s, which is an interesting issue for those researching tumors. According to some reports, SMA exhibited stronger anti-angiogenic activity than MA and showed suppression of tumor growth and tumor-induced angiogenesis in an MDA-MB-231 human breast cancer cell-inoculated CAM assay [22]. The main mechanism of this study was that SMA and MA mediated the inhibition of class I PI3K activity and signaling to inhibit the angiogenic effect.

Recently, many papers on chemotherapy treatment, especially anti-angiogenesis, were published [11, 14–16, 28]. The main targets of these papers were cilengitide (an anti-angiogenic inhibitor of integrins, αvβ3 and αvβ5) and bevacizumab (an anti-angiogenic inhibitor of vascular endothelial growth factor A). For instance, cilengitide was used in combination therapy with belotecan against glioblastoma [11]. The authors used immunohistochemistry to confirm the anti-angiogenic effect with CD 31, a marker of endothelial cells and tumor angiogenesis and used *in vitro* and *in vivo* experiments to confirm anti-tumor effects and combination therapy with cilengitide and belotecan to prolong the survival of mice. These results showed a favorable expectation for the treatment of glioblastoma. In a multicenter randomized open-label phase 3 trial for cilengitide combined with standard treatment, however, they demonstrated dismal results [15]. Patients did not gain any benefits from cilengitide, the median overall survival in the control group was the same as the cilengitide group. The results of clinical trials were not as good as the laboratory data, in reality. The results of SMA salt from the lab experiments were attractive for researching GBM treatment. Therefore, the variable effects of SMA salt on GBM cell lines should be validated with GBM patients in the further clinical studies.

In conclusion, this study has shown the feasibility of combination therapy on GBM cell lines *in vitro* and *in vivo*. Although the effect of combination therapy in survival analysis is not as satisfactory as our previous study, we might suggest that the effects of combination therapy with SMA salt and TMZ attributed to the inhibition of migration and invasion activities and anti-tumor effects on GBM cell lines. SMA salt could be one of the promising candidates for combination therapy in clinical settings. The exact mechanisms of combination therapy of TMZ and SMA salt in GBM cell lines will need more examination in future studies.

## MATERIALS AND METHODS

### Cell lines

The U87MG, U251MG, and LN229 cell lines were human glioma cell lines purchased from American Type Culture Collection (ATCC, Manassas, VA, USA). Cells were cultured in complete Dulbecco's Modified Eagle Medium (DMEM), which was supplemented with 10% fetal bovine serum (FBS) and incubated at 37°C and 5% CO_2_.

### Cell viability assay

The cytotoxicity of SMA salt (Daewoo Pharmaceutical Company, Seoul, Korea), TMZ and a combination of SMA salt and TMZ was measured using the Cell Counting Kit-8 (CCK-8; Dojindo Molecular Technologies Inc., Tokyo, Japan). Each cell line was seeded into 96-well plates at a density of 5 × 10^3^ cells per well to allow for overnight adhesion. The next day, the cells were treated with SMA salt at a concentration of 25 and 50 μM and TMZ at a concentration of 500 μM. After 2 days, we added 10 μL of the CCK-8 solution per well and then incubated for 2 hrs at 37°C and 5% CO_2_. The optical density (OD) of the sample plate was measured at 450 nm by an ELISA reader (VERSAmax microplate reader, Molecular Device, CA, USA). The viability of the tumor cells was assessed by calculating the OD ratio of the specific OD in each sample to the OD of the control sample.

### Cell migration assay

The insert of a 24-well Transwell apparatus (Corning, Corning, NY) was incubated at 37°C for 1-2 hrs. The U87MG, U251MG and LN229 cells (2 × 10^5^ cells/mL) were treated with SMA salt, TMZ, and the combination of SMA salt and TMZ and prepared in serum-free medium. FBS-containing medium (750 μL) was added to the lower chamber and 200 μL of prepared cell suspension was added to the insert. After 24 hrs, cells that were remaining in the insert (i.e., non-invading cells) were gently retrieved using a cotton-tipped swab and allowed to air dry for 20 min. A solution of 0.4% crystal violet (500 μL) was added to each well of the apparatus. After 10 min, the migrated cells that traversed the membrane separating the insert from the lower chamber were stained by dipping the lower surface of the membrane into the stain. The stained membranes were washed several times with water and allowed to air dry. The results of cell migration assays are described as a percentage (count in the lower chamber as % of total cell count).

### Cell invasion assay

Matrigel (BD, Franklin Lakes, NJ) was thawed overnight at 4 ng and diluted (1-5 mg/mL) in serum-free cold DMEM. A 100 μL volume of the diluted Matrigel was added to the upper chamber of a Transwell apparatus and incubated at 37°C for 4-5 hrs to allow the gel to swell. The treated U87MG, U251MG and LN229 cells (2 × 10^5^ cells/mL) were prepared in serum-free medium, and 200 μL of prepared cell suspension was added to each insert. Medium containing FBS (750 μL) was added to the lower chamber of the Transwell apparatus. After 24 hrs, non-invading cells were removed, and the invading cells were quantified as described above [29].

### Orthotopic mouse model

Six-week-old Female Balb/c-nu mice were ordered from Orient Bio (Seongnam-si, Korea; distributor for Charles River, Wilmington, MA). Thirty mice were used for tumor volume and survival analysis. Each experiment utilized 15 mice. Fifteen mice were divided into 3 groups in each experiment. All of them were anesthetized and the head was fixed in a stereotactic frame, followed by the creation of a midline scalp incision (1 cm). A small hole was made 0.5 mm anterior and 2 mm lateral from the bregma. A sterile 10 μL Hamilton syringe with a #26S needle was inserted at a depth of 3.5 mm from the surface of the skull and withdrawn by 0.5 mm to inject 2 × 10^5^ U87MG cells in a volume of 2 μL. The injection rate was 0.5 mL/min. After the implantation of the tumor cells, the needle was kept in place for 3 min to prevent cell reflux. Then, the needle was completely withdrawn from the brain over the course of 3 min (1.0 mm/min). Finally, the hole was sealed and the skin was sutured. All of these steps were followed in our lab protocol. This study was approved by the Institutional Animal Care and Use Committee of the Medical Science Research Institute, Seoul National University Bundang Hospital (authorization number: BA1411-164/058-01).

### Treatment protocol and survival analysis

The orthotopic mice were randomly divided into three groups: a control group, TMZ group and combination group (SMA salt and TMZ). The mice in the control group were injected with saline only, and both SMA salt and TMZ were administered at a dose of 50 mg/kg daily intraperitoneally in the TMZ and combination groups. The drug treatments began 7 days after the implantation of tumor cells and for 5 days per week. Half of the mice were sacrificed 5 weeks after the implantation of the tumor cells for tumor volume analysis; the remaining mice were observed for another 3 months to analyze survival. Death was defined as a weight reduction to 75% of their initial weight or an unexpected sudden death before that weight was reached.

### Evaluation of tumor growth

When we obtained mice brains, the mice were perfused with phosphate-buffered saline (PBS) and then fixed in 4% paraformaldehyde; after that, the brain was removed for paraffin embedding. The fixed brains were coronally sectioned into slices of 5 μm thickness. The slices were mounted and stained with hematoxylin and eosin. The maximal length (*L*), width (*W*) and height (*H*) of each tumor sample was measured and used the following formula to calculate the tumor volume [30].

### Statistical analysis

We used Student's *t*-test analysis for the statistical analysis of our data. The Kaplan–Meier method was used for the survival analysis. Differences in survival were tested for significance using the two-sided log-rank test. A value of P < 0.05 was considered significant. All analyses were performed using the SPSS statistical software package (released 17.0.1, 2008; SPSS, Chicago, IL).

## Abbreviations

SMA salt: 7-O-succinyl macrolactin A tromethamine salt
SMA: 7-O-succinyl macrolactin A
MA: macrolactin A
TMZ: temozolomide
GBM: glioblastoma
combination group: TMZ and SMA salt group
ATCC: American Type Culture Collection
DMEM: Dulbecco's Modified Eagle Medium
CCK-8: Cell Counting Kit-8
OD: optical density.
